# Symplectic Radon Transform and the Metaplectic Representation

**DOI:** 10.3390/e24060761

**Published:** 2022-05-28

**Authors:** Maurice A. de Gosson

**Affiliations:** Faculty of Mathematics (NuHAG), University of Vienna, Oskar-Morgenstern-Platz 1, 1090 Vienna, Austria; maurice.de.gosson@univie.ac.at

**Keywords:** radon transform, metaplectic group, Lagrangian planes, symplectic tomography

## Abstract

We study the symplectic Radon transform from the point of view of the metaplectic representation of the symplectic group and its action on the Lagrangian Grassmannian. We give rigorous proofs in the general setting of multi-dimensional quantum systems. We interpret the Radon transform of a quantum state as a generalized marginal distribution for its Wigner transform; the inverse Radon transform thus appears as a “demarginalization process” for the Wigner distribution.

## 1. Introduction

The idea of using what is today called the “Radon transform” to reconstruct a function from partial data goes back to the 1917 work [1] by the Austrian mathematician Johann Radon. While Radon originally only considered two or three-dimensional systems (in which case it is called the “X-ray transform”), the theory has since then been generalized to arbitrary Euclidean spaces. The applications of the Radon transform to quantum mechanics and optics have been developing rapidly these last years (see the paper [2] by Vogel and Risken). Using an approach from Mancini, Man’ko, and Tombesi [3], several authors [4,5,6,7,8,9,10] study what they call the “symplectic Radon transform” of a mixed quantum state; in a recent paper [11], some of these results are extended to the framework of $C^{*}$ algebras. The aim of the present paper is to give a simple rigorous approach to the theory of the symplectic Radon transform in several degrees of freedom. For this, we will use systematically the theory of the metaplectic group as developed in our previous work [12], together with the elementary theory of Lagrangian subspaces of the standard symplectic space. Our main observation is the following: integration of the Wigner transform $W_{\psi }\left(x,p\right)$ (of a function, or of a state) along the *x* and *p* coordinate planes give the correct probability distributions ${\left|\psi \left(x\right)\right|}^{2}$ and $|\hat{\psi }{\left(p\right)|}^{2}$ in position and momentum space). However, these are not sufficient to reconstruct the state ψ (this is an aspect of the “Pauli problem”, see Section 4). However, applying a metaplectic transform $\hat{U}$ associated with a symplectic rotation *U* to the state ψ transforms the Wigner transform following the rule $W_{\hat{U}\psi }\left(x,p\right)=W_{\psi }\left(U^{-1}\left(x,p\right)\right)$ (this is the “symplectic covariance property” of the Wigner transform, which is well-known in harmonic analysis, especially in the Weyl–Wigner–Moyal approach to quantum mechanics). This use of metaplectic transforms leads, by calculating the marginals of $W_{\hat{U}\psi }\left(x,p\right)$ along the *x* and *p* coordinate planes to infinitely many probability distributions, and these allow the reconstruction of the state. More precisely, we will redefine the Radon transform as being given by the formula

$$R_{\psi }\left(X,A,B\right)=\det\Lambda^{-1}{\left|{\hat{U}}_{A,B}\psi \left(\Lambda^{-1}X\right)\right|}^{2}$$

where $X\in R^{n}$ and ${\hat{U}}_{A,B}$ is the metaplectic operator associated with the symplectic rotation

$$U_{AB}=\left(\begin{array}{cc} \Lambda^{-1}A & \Lambda^{-1}B\\ -\Lambda^{-1}B & \Lambda^{-1}A \end{array}\right)$$

where A,B are square matrices with rank(A,B)=n and $\Lambda ={\left(A^{T}A+B^{T}B\right)}^{1/2}$. We thereafter prove (under suitable conditions on ψ) the inversion formula

$$W_{\psi }\left(x,p\right)={\left(2\pi \hbar \right)}^{-2n^{2}}\int R_{\psi }\left(X,A,B\right)e^{\frac{i}{\hbar }\left(X-Ax-Bp\right)}dXdAdB$$

which reduces for n=1 to the usual inversion formula found in the literature (the case n=1 is discussed in Section 2 to motivate the results in the general case). As an illustration, we apply our constructions to the generalized Gaussian states

(1) $$\psi_{V,W}\left(x\right)={\left(\frac{1}{\pi \hbar }\right)}^{n/4}{\left(\det V\right)}^{1/4}e^{-\frac{1}{2\hbar }\left(V+iW\right)x^{2}}\;.$$

in Section 3, which gives us the opportunity to shortly discuss the Pauli problem for Gaussians, thus generalizing results in [13].

This paper is structured as follows:

- In Section 2, we study the “easy case” of systems with one degree of freedom; this allows us to present the main ideas of this paper without using too much new terminology (for instance, any line through the origin of the phase plane is a Lagrangian subspace).
- In Section 3, we deal with the multi-dimensional case and give a working definition of the symplectic Radon transform from the point of view of operator theory, with a particular emphasis on the metaplectic representation of the symplectic group. We prove an inversion formula allowing the reconstruction of a state from the knowledge of its Radon transform; in particular, the case of Gaussian states is discussed, which allows to give new insight in the old “Pauli problem”.
- For the reader’s convenience, we have included two appendices where we shortly review the main properties of the metaplectic group (Appendix A) and of the Lagrangian Grassmannian (Appendix B).

## 2. The Case *n* = 1

Let $\hat{\rho }$ be a mixed quantum state with one degree of freedom: $\hat{\rho }$ is a positive-semidefinite trace class operator on $L^{2}\left(R\right)$ with trace $\mathrm{Tr}(\hat{\rho })=1$. In view of the spectral theorem, there exists a sequence $\left(\psi_{j}\right)$ with $\psi_{j}\in L^{2}\left(R\right)$ and a sequence of non-negative numbers $\left(\lambda_{j}\right)$ with $\sum_{j}\lambda_{j}=1$ such that $\hat{\rho }=\sum_{j}\lambda_{j}|\psi_{j}\rangle \langle \psi_{j}|$ where $|\psi_{j}\rangle \langle \psi_{j}|$ is the orthogonal projection of $L^{2}\left(R\right)$ onto the ray $C\psi_{j}$. By definition [14,15], the Wigner distribution of $\hat{\rho }$ is the convex sum

(2) $$\rho =\sum_{j}\lambda_{j}W_{\psi j}$$

where $W_{\psi j}$ is the usual Wigner transform of $\psi_{j}$, defined for $\psi \in L^{2}\left(R\right)$ by

(3) $$W_{\psi }\left(x,p\right)=\frac{1}{2\pi \hbar }\int_{-\infty }^{\infty }e^{-\frac{i}{\hbar }py}\psi \left(x+\frac{1}{2}y\right)\psi^{*}\left(x-\frac{1}{2}y\right)dy.$$

Recall [14,15,16] that the marginal properties

(4) $$\int_{-\infty }^{\infty }W_{\psi }\left(x,p\right)dp={\left|\psi \left(x\right)\right|}^{2}\;\;,\;\;\int_{-\infty }^{\infty }W_{\psi }\left(x,p\right)dx={\left|\hat{\psi }\left(p\right)\right|}^{2}$$

make sense provided that ψ and its Fourier transform are, in addition to being square integrable, absolutely integrable: $\psi ,\hat{\psi }\in L^{1}\left(R\right)$.

In most texts studying the tomographic picture of quantum mechanics, the symplectic Radon transform of the quantum state $\hat{\rho }$ is defined by the integral

(5) $$R_{\hat{\rho }}\left(X,a,b\right)=\int \rho \left(x,p\right)\delta \left(X-ax-bp\right)dpdx$$

where *a* and *b* are real numbers, and it is claimed that the following essential reconstruction formula holds

(6) $$\rho \psi \left(x,p\right)=\frac{1}{2\pi \hbar }\int R_{\hat{\rho }}\left(X,a,b\right)e^{\frac{i}{\hbar }\left(X-ax-bp\right)}dXdadb.$$

The following result is simultaneously a rigorous restatement and a justification of these formulas. It will be extended to the case of quantum states with an arbitrary number *n* of freedom in the forthcoming sections. Among other things, we see that the inverse Radon transform can be viewed as a “demarginalization process” [17] for the Wigner distribution.

We will use the following notation: we set $U_{a,b}=\left(\begin{array}{cc} a/\lambda & b/\lambda \\ -b/\lambda & a/\lambda \end{array}\right)$ where $\lambda =\sqrt{a^{2}+b^{2}}$; clearly, $U_{a,b}$ is a rotation in the x,p plane.

**Theorem** **1.** *Let $\hat{\rho }$ be a pure quantum state: $\rho =2\pi \hbar W_{\psi }$ for some $\psi \in L^{2}\left(R\right)$. We assume that in addition $\psi ,\hat{\psi }\in L^{1}\left(R\right)$, which ensures that the marginal properties are satisfied.*

*(i)*  *The Radon transform $R_{\hat{\rho }}\left(X,a,b\right)$ is given by the formula*
(7) $$R_{\hat{\rho }}\left(X,a,b\right)=\lambda^{-1}{\left|{\hat{U}}_{a,b}\psi \left(\lambda^{-1}X\right)\right|}^{2}$$
  *where ${\hat{U}}_{a,b}\in \mathrm{Mp}\left(n\right)$ is anyone of the two metaplectic operators covering the rotation $U_{a,b}$.*
*(ii)*  *The inverse Radon transform is given by the formula:*
(8) $$W_{\psi }\left(x,p\right)=\frac{1}{2\pi \hbar }\int R_{\hat{\rho }}\left(X,a,b\right)e^{\frac{i}{\hbar }\left(X-ax-bp\right)}dXdadb.$$
*(iii)*  *The Radon transform of ψ is given by the line integral*
(9) $$R_{\hat{\rho }}\left(X,a,b\right)=\int_{-\infty }^{\infty }W_{\psi }\left(z\left(t\right)\right)\left|\dot{z}\left(t\right)\right|dt$$
  *where t⟼z(t) is a parametrization of the straight line $\ell_{a,b}^{X}$ in $R^{2}$ with equation ax+bp=X.*

**Proof.**  It is sufficient to assume that $\hat{\rho }$ is a pure state, that is, $\rho =2\pi \hbar W_{\psi }$ for some ψ. (i) Let us make the change of variables

(10) $$\left(\begin{array}{c} u\\ v \end{array}\right)=\left(\begin{array}{cc} a/\lambda & b/\lambda \\ -b/\lambda & a/\lambda \end{array}\right)\left(\begin{array}{c} x\\ p \end{array}\right)$$

in the integral (5). This leads to the expression

(11) $$R_{\hat{\rho }}\left(X,a,b\right)=\;\int \int \;W_{\psi }\left(U_{a,b}^{-1}\left(u,v\right)\right)\delta \left(X-\lambda u\right)dudv.$$

Since $\delta \left(X-\lambda u\right)=\lambda^{-1}\delta \left(\lambda^{-1}X-u\right)$, this can be rewritten

(12) $$R_{\hat{\rho }}\left(X,a,b\right)=\lambda^{-1}\;\int \int \;W_{\psi }\left(\lambda^{-1}\left(au-bv,bu+av\right)\right)\delta \left(\lambda^{-1}X-u\right)dudv.$$

In view of the symplectic covariance property [12,16,18] of the Wigner transform, we have

(13) $$W_{\psi }\left(U_{a,b}^{-1}\left(u,v\right)\right)=W_{{\hat{U}}_{a,b}\psi }\left(u,v\right)$$

where ${\hat{U}}_{a,b}$ is any one of the two metaplectic operators (see the Appendix A) covering *U* and hence (12) yields

$$\begin{array}{cc} R_{\hat{\rho }}\left(X,a,b\right) & =\lambda^{-1}\;\int \int \;W_{{\hat{U}}_{a,b}\psi }\left(\lambda^{-1}X,v\right)\delta \left(\lambda^{-1}X-u\right)dudv\\ \; & =\lambda^{-1}\int_{-\infty }^{\infty }W_{{\hat{U}}_{a,b}\psi }\left(\lambda^{-1}X,v\right)dv, \end{array}$$

hence Formula (7) using the marginal properties (4). (ii) Let us denote *A* the right-hand side of the equality (8). Using the first marginal property (4), we have

$$\begin{array}{cc} A & =\lambda^{-1}\frac{1}{2\pi \hbar }\int_{R^{3}}{\left|{\hat{U}}_{a,b}\psi \left(\lambda^{-1}X\right)\right|}^{2}e^{\frac{i}{\hbar }\left(X-ax-bp\right)}dXdadb\\ \; & =\lambda^{-1}\frac{1}{2\pi \hbar }\int_{R^{4}}W_{{\hat{U}}_{a,b}\psi }\left(\lambda^{-1}X,P\right)e^{\frac{i}{\hbar }\left(X-ax-bp\right)}dXdPdadb. \end{array}$$

Replacing *X* with λX and using the symplectic covariance property (13), we get

$$\begin{array}{cc} A & =\frac{1}{2\pi \hbar }\int_{R^{4}}WW_{{\hat{U}}_{a,b}\psi }\left(X,P\right)e^{\frac{i}{\hbar }\left(\lambda X-ax-bp\right)}dXdPdadb\\ \; & =\frac{1}{2\pi \hbar }\int_{R^{4}}W_{\psi }\left(U_{a,b}^{-1}\left(X,P\right)\right)e^{\frac{i}{\hbar }\left(\lambda X-ax-bp\right)}dXdPdadb\\ \; & =\frac{1}{2\pi \hbar }\int_{R^{4}}W_{\psi }\left(\left(a/\lambda \right)X-\left(b/\lambda \right)P,\left(b/\lambda \right)X+\left(a/\lambda \right)P\right))e^{\frac{i}{\hbar }\left(\lambda X-ax-bp\right)}dXdPdadb. \end{array}$$

Setting Y=(a/λ)X−(b/λ)P and Z=(b/λ)X+(a/λ)P (and hence λX=aY+bZ), we have dXdP=dYdZ so that

$$A=\frac{1}{2\pi \hbar }\int_{R^{4}}W_{\psi }\left(Y,Z\right))e^{\frac{i}{\hbar }\left(a\left(Y-x\right)+b\left(Z-p\right)\right)}dYdZdadb.$$

In view of the Fourier inversion formula, written formally as

$$\;\int \int \;e^{\frac{i}{\hbar }\left(a\left(Y-x\right)+b\left(Z-p\right)\right)}dadb=2\pi \hbar \delta \left(Y-x,Z-p\right)$$

we thus have

$$A=\;\int \int \;W_{\psi }\left(x,p\right)\delta \left(Y-x,Z-p\right)dYdZ=W_{\psi }\left(x,p\right)$$

which was to be proven. (iii) It is sufficient to show that (9) holds for one parametrization. In view of Formula (11), we have

(14) $$\begin{array}{cc} R_{\hat{\rho }}\left(X,a,b\right) & =\lambda^{-1}\;\int \int \;W_{\psi }\left(U_{a,b}^{-1}\left(u,v\right)\right)\delta \left(\lambda^{1}X-u\right)dudv \end{array}$$

(15) $$\begin{array}{cc} \; & =\lambda^{-1}\int_{-\infty }^{\infty }\left(\int_{-\infty }^{\infty }W_{\psi }\left(U_{a,b}^{-1}\left(u,v\right)\right)\delta \left(\lambda^{1}X-u\right)du\right)dv \end{array}$$

(16) $$\begin{array}{cc} \; & =\int_{-\infty }^{\infty }W_{\psi }\left(U_{a,b}^{-1}\left(\lambda^{-1}X,v\right)\right)dv \end{array}$$

that is, since $U_{a,b}^{-1}=\left(\begin{array}{cc} a/\lambda & -b/\lambda \\ b/\lambda & a/\lambda \end{array}\right)$, and replacing *v* with *t*,

(17) $$R_{\hat{\rho }}\left(X,a,b\right)=\int_{-\infty }^{\infty }W_{\psi }\left(a\lambda^{-2}X-v\lambda^{-1}t,v\lambda^{-2}X+a\lambda^{-1}t\right)dt.$$

Set now $x\left(t\right)=a\lambda^{-2}X-v\lambda^{-1}t$ and $p\left(t\right)=v\lambda^{-2}X+a\lambda^{-1}t$. Then, ax(t)+bp(t)=X and $\dot{x}{\left(t\right)}^{2}+\dot{p}{\left(t\right)}^{2}=1$; hence, (17) implies (9). □

**Remark** **1.** *The first part of the theorem above uses the physicist’s definition (5) and can thus be taken as a mathematically correct redefinition of the Radon transform. We will exploit this fact in the next section.*

## 3. The Multivariate Case

For n≥1, we consider $R^{2n}\equiv T^{*}R^{n}$ equipped with its standard symplectic structure, which is defined by

$$\sigma \left(z,z^{\prime }\right)=Jz\cdot z^{\prime }\;\;,\;\;J=\left(\begin{array}{cc} 0 & I\\ -I & 0 \end{array}\right).$$

We denote by Sp(n) the symplectic group of $\left(R^{2n},\sigma \right)$ and by Mp(n) its unitary representation of its double cover (the metaplectic group; see Appendix A).

### 3.1. Definitions

Let A,B be two real square n×n matrices with such that $A^{T}B=BA^{T}$ and rank(A,B)=n. Setting

$$M_{AB}=\left(\begin{array}{cc} A & B\\ -B & A \end{array}\right)\;\;,\;\;\Lambda ={\left(A^{T}A+B^{T}B\right)}^{1/2}$$

and noting that $A^{T}A+B^{T}B$ is invertible, we have the factorization

$$M_{AB}=\left(\begin{array}{cc} \Lambda & 0\\ 0 & \Lambda \end{array}\right)\left(\begin{array}{cc} \Lambda^{-1}A & \Lambda^{-1}B\\ -\Lambda^{-1}B & \Lambda^{-1}A \end{array}\right)$$

where

(18) $$U_{A,B}=\left(\begin{array}{cc} \Lambda^{-1}A & \Lambda^{-1}B\\ -\Lambda^{-1}B & \Lambda^{-1}A \end{array}\right)\in U\left(n\right)$$

is a symplectic rotation. Note that its inverse is

(19) $$U_{A,B}^{-1}=\left(\begin{array}{cc} A^{T}\Lambda^{-1} & -B^{T}\Lambda^{-1}\\ -B^{T}\Lambda^{-1} & A^{T}\Lambda^{-1} \end{array}\right).$$

**Definition** **1.** *The symplectic Radon transform of ψ is the transformation*

$$R_{\psi }\left(\cdot ,A,B\right):L^{2}\left(R^{n}\right)⟶L^{1}\left(R^{n}\right)$$

*defined, for $\psi \in L^{2}\left(R^{n}\right)$ by*

(20) $$R_{\psi }\left(X,A,B\right)=\det\Lambda^{-1}{\left|{\hat{U}}_{A,B}\psi \left(\Lambda^{-1}X\right)\right|}^{2}$$

*where $\Lambda ={\left(A^{T}A+B^{T}B\right)}^{1/2}$ and the unitary operator*

$${\hat{U}}_{A,B}:L^{2}\left(R^{n}\right)⟶L^{2}\left(R^{n}\right)$$

*is any of the two elements of Mp(n) covering the symplectic rotation $U_{A,B}$.*

That $R_{\psi }\left(\cdot ,A,B\right)$ maps $L^{2}\left(R^{n}\right)$ into $L^{1}\left(R^{n}\right)$ is clear, since ${\hat{U}}_{A,B}$ is unitary on $L^{2}\left(R^{n}\right)$.

When detB≠0, the metaplectic operator ${\hat{U}}_{A,B}$ is defined by

(21) $$\begin{array}{cc} {\hat{U}}_{A,B}\psi \left(x\right) & ={\left(\frac{1}{2\pi \hbar }\right)}^{n/2}i^{m-n/2}\sqrt{|\det B^{-1}|}\int_{R^{n}}e^{\frac{i}{\hbar }A\left(x,x^{\prime }\right)}\psi \left(x^{\prime }\right)dx^{\prime } \end{array}$$

(22) $$\begin{array}{cc} A(x,x^{\prime }) & =\frac{1}{2}AB^{-1}x\cdot x-B^{-1}x\cdot x^{\prime }+\frac{1}{2}B^{-1}Ax^{\prime }\cdot x^{\prime } \end{array}$$

after one has made a choice of the integer *m* modulo 4 (see Appendix A).

Recall that in the multi-dimensional case, the Wigner transform $W_{\psi }$ of $\psi \in L^{2}\left(R^{n}\right)$ is given by the integral

(23) $$W_{\psi }\left(x,p\right)={\left(\frac{1}{2\pi \hbar }\right)}^{n}\int_{R^{n}}e^{-\frac{i}{\hbar }py}\psi \left(x+\frac{1}{2}y\right)\psi^{*}\left(x-\frac{1}{2}y\right)dy$$

and that the marginal properties generalizing (4) hold

(24) $$\int_{R^{n}}W_{\psi }\left(x,p\right)dp={\left|\psi \left(x\right)\right|}^{2}\;\;,\;\;\int_{R^{n}}W_{\psi }\left(x,p\right)dx={\left|\hat{\psi }\left(p\right)\right|}^{2}$$

for $\psi ,\hat{\psi }\in L^{1}\left(R^{n}\right)$; the Fourier transform $\hat{\psi }=F\psi$ is here given by

(25) $$F\psi \left(p\right)={\left(\frac{1}{2\pi \hbar }\right)}^{n/2}\int_{R^{n}}e^{-\frac{i}{\hbar }p\cdot x}\psi \left(x\right)dx.$$

### 3.2. The Radon Inversion Formula

We have the following straightforward generalization of the inversion result (ii) in Theorem 1:

**Theorem** **2.** *Viewing A and B as elements of $R^{n^{2}}$, we have*

(26) $$W_{\psi }\left(x,p\right)={\left(2\pi \hbar \right)}^{-2n^{2}}\int_{R^{n(2n+1)}}R_{\psi }\left(X,A,B\right)e^{\frac{i}{\hbar }\left(X-Ax-Bp\right)}dXdAdB$$

*where $dA=\prod_{i,j=1}^{n}da_{ij}$ and $dB=\prod_{i,j}^{n}db_{ij}$ if $A={\left(a_{ij}\right)}_{1\leq i,j\leq n}$, $B={\left(b_{ij}\right)}_{1\leq i,j\leq n}$.*

**Proof.**  It goes exactly as the proof of Theorem 1 (ii). Let us denote by *W* the integral in the right-hand side of (26). We have, using the first marginal condition (24),

$$\begin{array}{cc} W & =\det\Lambda^{-1}\int {\left|{\hat{U}}_{A,B}\psi \left(\Lambda^{-1}X\right)\right|}^{2}e^{\frac{i}{\hbar }\left(X-Ax-Bp\right)}dXdAdB\\ \; & =\det\Lambda^{-1}\int W\left({\hat{U}}_{A,B}\psi \right)\left(\Lambda^{-1}X,P\right)e^{\frac{i}{\hbar }\left(X-Ax-Bp\right)}dPdXdAdB\\ \; & =\int W\left(\psi \right)\left(U_{A,B}^{-1}\left(X,P\right)\right)e^{\frac{i}{\hbar }\left(\Lambda X-Ax-Bp\right)}dPdXdAdB\\ \; & =\int W\left(\psi \right)\left(U_{A,B}^{-1}\left(X,P\right)\right)e^{\frac{i}{\hbar }\left(\Lambda X-Ax-Bp\right)}dPdXdAdB, \end{array}$$

that is, by Formula (19):

$$\begin{array}{c} W=\int W\left(\psi \right)\left(A^{T}\Lambda^{-1}X-B^{T}\Lambda^{-1}P,B^{T}\Lambda^{-1}X+A^{T}\Lambda^{-1}P\right))\\ \times e^{\frac{i}{\hbar }\left(\Lambda X-Ax-Bp\right)}dPdXdAdB. \end{array}$$

Setting $Y=A^{T}\Lambda^{-1}X-B^{T}\Lambda^{-1}P$ and $Z=B^{T}\Lambda^{-1}X+A^{T}\Lambda^{-1}P)$, we have dYdZ=dPdX and hence

$$W=\int W_{\psi }\left(Y,Z\right)e^{\frac{i}{\hbar }\left(A\left(Y-x\right)+B\left(Z-p\right)\right)}dYdZdAdB.$$

Integration of the exponential with respect to the variables *A* and *B* yields

$$\int_{R^{2n^{2}}}e^{\frac{i}{\hbar }\left(A\left(Y-x\right)+B\left(Z-p\right)\right)}dAdB={\left(2\pi \hbar \right)}^{2n^{2}}$$

and hence

$$\begin{array}{cc} W & ={\left(2\pi \hbar \right)}^{2n^{2}}\int W_{\psi }\left(Y,Z\right)\delta \left(Y-x,Z-p\right)|dYdZ\\ \; & ={\left(2\pi \hbar \right)}^{2n^{2}}W_{\psi }\left(x,p\right) \end{array}$$

which was to be proven. □

### 3.3. Interpretation as Generalized Marginals

Let us return to the definition (20) of the Radon transform:

$$R_{\psi }\left(X,A,B\right)=\det\Lambda^{-1}{\left|{\hat{U}}_{A,B}\psi \left(\Lambda^{-1}X\right)\right|}^{2}.$$

If we choose A=I and B=0, this reduces to the formula

$$R_{\psi }\left(X,I,0\right)={\left|\psi \left(X\right)\right|}^{2}=\int_{R^{n}}W_{\psi }\left(X,P\right)dP;$$

similarly, if A=0 and B=I, we get

$$R_{\psi }\left(X,A,B\right)={\left|F\psi \left(X\right)\right|}^{2}=\int_{R^{n}}W_{\psi }\left(P,X\right)dX.$$

definition (20) reduces to the formula

$$R_{\psi }\left(X,A,B\right)={\left|{\hat{U}}_{A,B}\psi \left(X\right)\right|}^{2}$$

showing that the Radon transform is essentially a margin property for the “rotated” Wigner transform of ψ. In fact, we can view $R_{\psi }\left(X,A,B\right)$ as the surface integral of the Wigner transform on the (affine) Lagrangian plane (see Appendix B)

$$\ell_{A,B}^{X}=\left\{\left(x,p\right)\in R^{2n}:Ax+Bp=X\right\}.$$

In view of the marginal properties and the symplectic covariance of the Wigner transform, followed by the change of variables $P⟼\Lambda^{-1}P$, we have

$$\begin{array}{cc} |{\hat{U}}_{A,B}\psi \left(\Lambda^{-1}X\right){|}^{2} & =\int_{R^{n}}W\left({\hat{U}}_{A,B}\psi \right)\left(\Lambda^{-1}X,P\right)dP\\ \; & =\int_{R^{n}}W_{\psi }\left(U_{A,B}^{-1}\left(\Lambda^{-1}X,P\right)\right)dP\\ \; & =\det\Lambda \int_{R^{n}}W_{\psi }\left(U_{A,B}^{-1}\left(\Lambda^{-1}X,\Lambda^{-1}P\right)\right)dP, \end{array}$$

that is, explicitly,

$$\begin{array}{cc} |{\hat{U}}_{A,B}\psi \left(\Lambda^{-1}X\right){|}^{2}=\det\Lambda & \\ & \times \int_{R^{n}}W_{\psi }\left(A^{T}\Lambda^{-1}X-B^{T}\Lambda^{-1}P,B^{T}\Lambda^{-1}X+A^{T}\Lambda^{-1}P\right)dP. \end{array}$$

Using the multi-parametrization

$$\begin{array}{cc} X^{\prime }\left(P\right) & =A^{T}\Lambda^{-1}X-B^{T}\Lambda^{-1}P\\ P^{\prime }\left(P\right) & =B^{T}\Lambda^{-1}X+A^{T}\Lambda^{-1}P \end{array}$$

we have

(27) $$R_{\psi }\left(X,A,B\right)=\int_{R^{n}}W_{\psi }\left(X^{\prime }\left(P\right),P^{\prime }\left(P\right)\right)dP$$

we have $AX^{\prime }\left(P\right)+BP^{\prime }\left(P\right)=X$, so we can interpret the formula above as a surface integral

$$|{\hat{U}}_{A,B}\psi \left(\Lambda^{-1}X\right){|}^{2}=\int_{\ell_{A,B}^{X}}W_{\psi }\left(Z\right)d\mu \left(Z\right)$$

where dμ(Z) is the Lebesgue measure on $\ell_{A,B}^{X}$.

## 4. Radon Transform of Generalized Gaussians

### 4.1. Generalized Gaussians

By generalized (centered) Gaussian, we mean a function

(28) $$\psi_{V,W}\left(x\right)={\left(\frac{1}{\pi \hbar }\right)}^{n/4}{\left(\det V\right)}^{1/4}e^{-\frac{1}{2\hbar }\left(V+iW\right)x^{2}}\;.$$

where *V* and *W* are real symmetric n×n matrices with V>0 (i.e., positive definite). Such centered Gaussians are generalizations of the usual squeezed coherent states appearing in the physical literature; see [12,18]. The function $\psi_{V,W}$ is normalized to unity: $\left|\right|\psi_{V,W}{\left|\right|}_{L^{2}}=1$ and its Wigner transform is given by

(29) $$W_{\psi V,W}\left(z\right)={\left(\frac{1}{\pi \hbar }\right)}^{n}e^{-\frac{1}{\hbar }Gz\cdot z}$$

where *G* is the symmetric and symplectic matrix

(30) $$G=\left(\begin{array}{cc} V+WV^{-1}W & WV^{-1}\\ V^{-1}W & V^{-1} \end{array}\right).$$

That G∈Sp(n) easily follows from the observation that $G=S^{T}S$ where

(31) $$S=\left(\begin{array}{cc} V^{1/2} & 0\\ V^{-1/2}W & V^{-1/2} \end{array}\right)$$

clearly is symplectic. Let ${\mathrm{Gauss}}_{0}\left(n\right)$ be the set of all centered Gaussians (28); a central (and often implicitly used) result is that the metaplectic group Mp(n) acts transitively on ${\mathrm{Gauss}}_{0}\left(n\right)$: we have an action

(32) $$\mathrm{Mp}\left(n\right)\times {\mathrm{Gauss}}_{0}\left(n\right)⟶{\mathrm{Gauss}}_{0}\left(n\right)$$

which is totally described by the action of $\hat{S}\in \mathrm{Mp}\left(n\right)$ on the fiducial coherent state [18]

(33) $$\phi_{0}\left(x\right)={\left(\frac{1}{\pi \hbar }\right)}^{n/4}e^{-\frac{1}{2\hbar }{\left|x\right|}^{2}}$$

whose Wigner transform is

(34) $$W\phi_{0}\left(z\right)={\left(\frac{1}{\pi \hbar }\right)}^{n}e^{-\frac{1}{\hbar }{\left|z\right|}^{2}}\;.$$

Using the symplectic covariance of the Wigner transform, it is not difficult to prove that if $\hat{S}$ covers the symplectic matrix $S=\left(\begin{array}{cc} A & B\\ C & D \end{array}\right)$, then

$$\hat{S}\phi_{0}\left(x\right)=e^{i\gamma }\psi_{V,W}\left(x\right)$$

where γ∈R and

$$\begin{array}{cc} U & ={\left(AA^{T}+BB^{T}\right)}^{-1}\\ V & =-\left(CA^{T}+DB^{T}\right){\left(AA^{T}+BB^{T}\right)}^{-1}. \end{array}$$

### 4.2. The Radon Transform of $\psi_{V,W}$

Let us calculate the Radon transform

(35) $$R_{\psi_{{}_{U,V}}}\left(X,A,B\right)=\left(\det\Lambda^{-1}\right){\left|{\hat{U}}_{A,B}\psi_{V,W}\left(\Lambda^{-1}X\right)\right|}^{2}$$

of $\psi_{V,W}$ using Formula (20). For this, we have to determine ${\hat{U}}_{A,B}\psi_{V,W}$, where ${\hat{U}}_{A,B}\in \mathrm{Mp}\left(n\right)$ covers the symplectic rotation

(36) $$U_{A,B}=\left(\begin{array}{cc} \Lambda^{-1}A & \Lambda^{-1}B\\ -\Lambda^{-1}B & \Lambda^{-1}A \end{array}\right).$$

The most natural (and easiest) way to determine ${\hat{U}}_{A,B}\psi_{V,W}$ is to use the symplectic covariance formula

$$W\left({\hat{U}}_{A,B}\psi_{V,W}\right)\left(z\right)=W_{\psi V,W}\left(U_{A,B}^{-1}z\right)$$

of the Wigner transform; it yields, taking (29) into account and using the relation $U_{A,B}^{-1}=U_{A,B}^{T}$

$$W\left({\hat{U}}_{A,B}\psi_{V,W}\right)\left(z\right)={\left(\frac{1}{\pi \hbar }\right)}^{n}e^{-\frac{1}{\hbar }U_{A,B}GU_{{}^{AB}}^{T}z\cdot z}.$$

To explicitly determine $G^{\prime }=U_{A,B}GU_{{}^{AB}}^{T}$ is a rather lengthy (though straightforward) calculation; however, since we have an action (32), we know (by (30)) that there will exist $V^{\prime }$ and $W^{\prime }$ such that

$$G^{\prime }=\left(\begin{array}{cc} V^{\prime }+W^{\prime }V^{\prime }{}^{-1}W^{\prime } & W^{\prime }V^{\prime }{}^{-1}\\ V^{\prime }{}^{-1}W^{\prime } & V^{\prime }{}^{-1} \end{array}\right)$$

corresponding to the Gaussian

$${\hat{U}}_{A,B}\psi_{V,W}=\psi_{V^{\prime },W^{\prime }}\left(x\right)={\left(\frac{1}{\pi \hbar }\right)}^{n/4}{\left(\det V^{\prime }\right)}^{1/4}e^{-\frac{1}{2\hbar }\left(V^{\prime }+iW^{\prime }\right)x^{2}}.$$

Now, since it is only the (squared) modulus of ${\hat{U}}_{A,B}\psi_{V,W}$ which appears in (35), we will have

$$|{\hat{U}}_{A,B}\psi_{V,W}{|}^{2}={\left(\frac{1}{\pi \hbar }\right)}^{n/2}{\left(\det V^{\prime }\right)}^{1/2}e^{-\frac{1}{2\hbar }V^{\prime }x^{2}}$$

so that it suffices to determine $V^{\prime }$, whose inverse is the lower right block of $G^{\prime }$. This is easily done using the relation $G^{\prime }=U_{A,B}GU_{{}^{AB}}^{T}$ and one finds, after a few calculations and simplifications,

(37) $$V^{\prime }=\Lambda {\left[BVB^{T}+\left(A-BW\right)V^{-1}{\left(A-BW\right)}^{T}\right]}^{-1}\Lambda .$$

Summarizing, we have, after insertion in (35),

(38) $$\begin{array}{cc} R_{\psi_{{}_{V,W}}}\left(X,A,B\right) & =C\\ \; & \times \exp\left[\left(-\frac{1}{2\hbar }{\left[BVB^{T}+\left(A-BW\right)V^{-1}{\left(A-BW\right)}^{T}\right]}^{-1}\right)X\cdot X\right] \end{array}$$

(39) $$C={\left(\frac{1}{\pi \hbar }\right)}^{n/2}{\left(\det V^{\prime }\right)}^{1/2}\det{\left[BVB^{T}+\left(A-BW\right)V^{-1}{\left(A-BW\right)}^{T}\right]}^{-1}.$$

Suppose for instance $\psi_{{}_{V,W}}$ is a “squeezed coherent state”; then, W=0 and $\psi_{V}=\psi_{V,0}$ is

$$\psi_{V}\left(x\right)={\left(\frac{1}{\pi \hbar }\right)}^{n/4}{\left(\det V\right)}^{1/4}e^{-\frac{1}{2\hbar }Vx^{2}}.$$

Its Radon transform is then

(40) $$\begin{array}{c} R_{\psi_{V}}\left(X,A,B\right)=C\exp\left[-\frac{1}{2\hbar }{\left(BVB^{T}+AV^{-1}A^{T}\right)}^{-1}X\cdot X\right] \end{array}$$

(41) $$\begin{array}{c} C={\left(\frac{1}{\pi \hbar }\right)}^{n/2}{\left(\det V^{\prime }\right)}^{1/2}\det{\left(BVB^{T}+AV^{-1}A^{T}\right)}^{-1} \end{array}$$

(42) $$\begin{array}{c} V^{\prime }=\Lambda {\left[BVB^{T}+AV^{-1}A^{T}\right]}^{-1}\Lambda \end{array}$$

### 4.3. Application: The Pauli Problem

When n=1, the Formula (38) takes the simple form (replacing A,B,V,W with scalars a,b,v,w)

(43) $$R_{\psi_{{}_{v,w}}}\left(X,a,b\right)=C\exp\left[-\frac{1}{2\hbar }{\left[b^{2}v+{\left(a-bw\right)}^{2}v^{-1}\right]}^{-1}X\cdot X\right]$$

where

$$\psi_{{}_{v,w}}\left(x\right)={\left(\frac{1}{\pi \hbar }\right)}^{1/4}v^{1/4}e^{-\frac{1}{2\hbar }\left(v+iw\right)x^{2}}.$$

Let us apply this formula to the “Pauli problem”. This problem goes back to the question Pauli asked in [19], whether the probability densities ${\left|\psi \left(x\right)\right|}^{2}$ and $|\hat{\psi }{\left(p\right)|}^{2}$ uniquely determine the wavefunction ψ(x). The answer is “no”: consider in fact the Gaussian wavepacket

(44) $$\psi \left(x\right)={\left(\frac{1}{2\pi \sigma_{xx}}\right)}^{1/4}e^{-\frac{x^{2}}{4\sigma_{xx}}}e^{\frac{i\sigma_{xp}}{2\hbar \sigma_{xx}}x^{2}}$$

whose Fourier transform of ψ is given by

(45) $$\hat{\psi }\left(p\right)=e^{i\gamma }{\left(\frac{1}{2\pi \sigma_{pp}}\right)}^{1/4}e^{-\frac{p^{2}}{4\sigma_{pp}}}e^{-\frac{i\sigma_{xp}}{2\hbar \sigma_{pp}}p^{2}}$$

where γ is an unessential constant real phase. Thus,

(46) $${\left|\psi \left(x\right)\right|}^{2}={\left(\frac{1}{2\pi \sigma_{xx}}\right)}^{1/2}e^{-\frac{x^{2}}{2\sigma_{xx}}}\;\;,\;\;{\left|\hat{\psi }\left(p\right)\right|}^{2}={\left(\frac{1}{2\pi \sigma_{pp}}\right)}^{1/2}e^{-\frac{p^{2}}{2\sigma_{pp}}}$$

and these relations imply the knowledge of $\sigma_{xx}$ and of $\sigma_{pp}$ but not of the covariance $\sigma_{xp}$ (the latter can actually be determined up to a sign using the fact that ψ saturates the Robertson–Schrödinger uncertainty principle: we have $\sigma_{xx}\sigma_{pp}-\sigma_{xp}^{2}=\frac{1}{4}\hbar^{2}$). Let us calculate the Radon transform of ψ using Formula (43). We have here $v=\hbar /2\sigma_{xx}$, $w=-\sigma_{xp}/\sigma_{xx}$; hence, Formula (43) becomes

(47) $$R_{\psi_{{}_{v,w}}}\left(X,a,b\right)=C\exp\left(-\frac{1}{2\hbar }{\left[\frac{b^{2}\hbar }{2\sigma_{xx}}+{\left(a+b\frac{\sigma_{xp}}{\sigma_{xx}}\right)}^{2}\frac{2\sigma_{xx}}{\hbar }\right]}^{-1}\right)X^{2}.$$

Notice that, as expected,

$$R_{\psi_{{}_{v,w}}}\left(X,1,0\right)={\left(\frac{1}{2\pi \sigma_{xx}}\right)}^{1/2}e^{-\frac{X^{2}}{4\sigma_{xx}}}={\left|\psi \left(x\right)\right|}^{2}$$

and, using the relation $\sigma_{xx}\sigma_{pp}-\sigma_{xp}^{2}=\frac{1}{4}\hbar^{2}$,

$$R_{\psi_{{}_{v,w}}}\left(X,0,1\right)={\left(\frac{1}{2\pi \sigma_{pp}}\right)}^{1/2}e^{-\frac{X^{2}}{2\sigma_{pp}}}={\left|\hat{\psi }\left(X\right)\right|}^{2}.$$

These relations show why we cannot recover the state ψ using the two radon transforms $R_{\psi_{{}_{v,w}}}\left(X,1,0\right)$ and $R_{\psi_{{}_{v,w}}}\left(X,0,1\right)$: none of them allow us to determine the covariance $\sigma_{xp}$. However, it suffices with one clever choice of the parameters *a* and *b* in Formula (47). Suppose indeed that we have measured, for some values of the parameters *a* and *b*, the positive quantity

(48) $$K=\frac{b^{2}\hbar }{2\sigma_{xx}}+{\left(a+b\frac{\sigma_{xp}}{\sigma_{xx}}\right)}^{2}\frac{2\sigma_{xx}}{\hbar }$$

appearing in the exponent of (47). Assuming that the variances $\sigma_{xx}$ and $\sigma_{pp}$ are known, we can find the covariance $\sigma_{xp}$ as follows: viewing (48) as a quadratic equation in the unknown $\sigma_{xp}$, we demand that this equation has exactly *one* real root. This imposes the relation

$$K=\frac{b^{2}\hbar }{2\sigma_{xx}}$$

and reduces (48) to the equation

$$a+b\frac{\sigma_{xp}}{\sigma_{xx}}=0$$

from which $\sigma_{xp}$ is unambiguously determined.

## 5. Concluding Remarks

In this paper, we outlined a novel approach to the symplectic Radon transform, which we believe is conceptually very simple once one has realized the fundamental role played in quantum mechanics by the metaplectic representation of the symplectic group. As we have discussed elsewhere [20] a few years ago, this is the shortest bridge between classical (Hamiltonian) mechanics, and its refinement, quantum mechanics. This being said, our approach is somewhat sketchy, since we have not characterized the classes of functions (or states) to which we can apply the radon transform, limiting ourselves, for simplicity, to the square integrable case. It is however well-known (at least by people belonging to the harmonic analysis community) that there are function spaces invariant under metaplectic transformations which are larger than the space of square integrable functions. We are, among other possibilities, thinking about Feichtinger’s modulation spaces [21], which are a very flexible tool for creating quantum states, and which can be used together with Shubin’s pseudodifferential calculus [22] to extend the theory (one could, for instance, envisage the reconstruction of general observables along these lines).

We will definitely come back to these topics in a near future.

## Data Availability

Not applicable.

## Appendix A. The Metaplectic Group Mp(n)

For a detailed study of the metaplectic group Mp(n) see [12,23]. For a rather “soft” (but still rigorous) approach, see [18].

Let $S=\left(\begin{array}{cc} A & B\\ C & D \end{array}\right)$ be a real 2n×2n matrix, where the “ blocks” A,B,C,D are n×n matrices. Let $J=\left(\begin{array}{cc} 0 & I\\ -I & 0 \end{array}\right)$ the standard symplectic matrix. We have S∈Sp(n) (the symplectic group) if and only $SJS^{T}=S^{T}JS=J$. These relations are equivalent to any of the two sets of conditions

(A1) $$\begin{array}{cc} A^{T}C,\;B^{T}D\;\;\mathrm{are}\;\mathrm{symmetric},\;\mathrm{and}\;A^{T}D-C^{T}B & =I \end{array}$$

(A2) $$\begin{array}{cc} AB^{T},\;CD^{T}\;\;\mathrm{are}\;\mathrm{symmetric},\;\mathrm{and}\;AD^{T}-BC^{T} & =I. \end{array}$$

One says that *S* is a *free symplectic matrix* if *B* is invertible, i.e., detB≠0. To a free symplectic matrix is associated a generating function: it is the quadratic form

(A3) $$A\left(x,x^{\prime }\right)=\frac{1}{2}DB^{-1}x\cdot x-B^{-1}x\cdot x^{\prime }+\frac{1}{2}B^{-1}Ax^{\prime }\cdot x^{\prime }.$$

The terminology comes from the fact that the knowledge of $A(x,x^{\prime })$ uniquely determines the free symplectic matrix *S*: we have

$$\left(\begin{array}{c} x\\ p \end{array}\right)=\left(\begin{array}{cc} A & B\\ C & D \end{array}\right)\left(\begin{array}{c} x^{\prime }\\ p^{\prime } \end{array}\right)⟺\left\{\begin{array}{c} p=\nabla_{x}A\left(x,x^{\prime }\right)\\ p^{\prime }=-\nabla_{x^{\prime }}A\left(x,x^{\prime }\right) \end{array}\right.$$

as can be verified by a direct calculation (the quadratic form A is thus a generating function of the free symplectic matrix *S*).

Now, to every free symplectic matrix $S_{A}$, we associate two operators ${\hat{S}}_{A,m}$ by the formula

(A4) $${\hat{S}}_{A,m}\psi \left(x\right)={\left(\frac{1}{2\pi \hbar }\right)}^{n/2}i^{m-n/2}\sqrt{|\det B^{-1}|}\int e^{\frac{i}{\hbar }A\left(x,x^{\prime }\right)}\psi \left(x^{\prime }\right)d^{n}x^{\prime }$$

where *m* (“Maslov index” [12]) corresponds to a choice of argument for $\det B^{-1}$: m=0 mod2 if $\det B^{-1}>0$ and m=1mod2 if $\det B^{-1}<0$ (*m* is defined modulo 4). It is not difficult to prove that the generalized Fourier transforms ${\hat{S}}_{A,m}$ are unitary operators on $L^{2}\left(R^{n}\right)$. These operators generate a group, the metaplectic group Mp(n). One shows that every $\hat{S}\in \mathrm{Mp}\left(n\right)$ can be written (non uniquely) as a product ${\hat{S}}_{A,m}{\hat{S}}_{A^{\prime },m^{\prime }}$. This group is a double covering of Sp(n), the covering projection being defined by

(A5) $$\pi_{\mathrm{Mp}}:\mathrm{Mp}\left(n\right)⟶\mathrm{Sp}\left(n\right)\;\;,\;\;\pi_{\mathrm{Mp}}\left({\hat{S}}_{A,m}\right)=S_{A}.$$

## Appendix B. The Lagrangian Grassmannian

A linear subspace *ℓ* of the symplectic space $\left(R^{2n},\sigma \right)$ is called a Lagrangian subspace (or plane) if it is maximally isotropic for the skew orthogonality relation $\sigma (z,z^{\prime })=0$. It must thus have dimension dimℓ=n and σ vanishes identically on *ℓ*. The set of all Lagrangian planes in $R^{2n}$ is called the Lagrangian Grassmannian and is denoted by Lag(n). The symplectic group Sp(n) acts transitively on Lag(n); in fact, the action

(A6) Sp(n)×Lag(n)⟶Lag(n)

thus defined induces a transitive action

(A7) U(n)×Lag(n)⟶Lag(n)

where U(n) is the image in Sp(n) of the unitary group U(n,C) by the monomorphism ι:u⟼U defined by $U\left(x,p\right)=\left(x^{\prime },p^{\prime }\right)$ if $u\left(x+ip\right)=x^{\prime }+ip^{\prime }.$ In matrix notation

$$\iota \left(A+iB\right)=\left(\begin{array}{cc} A & B\\ -B & A \end{array}\right).$$

The conditions (A1) and (A2) are here equivalent to

(A8) $$\begin{array}{cc} A^{T}B\;\mathrm{is}\;\mathrm{symmetric},\;\mathrm{and}\;A^{T}A+B^{T}B & =I \end{array}$$

(A9) $$\begin{array}{cc} AB^{T}\;\mathrm{is}\;\mathrm{symmetric},\;\mathrm{and}\;AA^{T}+BB^{T} & =I. \end{array}$$

The elements of U(n) are the symplectic rotations of $\left(R^{2n},\omega \right)$:

(A10) U(n)=Sp(n)∩O(2n,R)

and the transitivity of the action (A7) follows: let $\left(\ell ,\ell^{\prime }\right)\in \mathrm{Lag}\left(n\right)\times \mathrm{Lag}\left(n\right)$ and choose two bases $\left\{e_{1},\ldots ,e_{n}\right\}$ and $\left\{e_{1}^{\prime },\ldots ,e_{n}^{\prime }\right\}$ of *ℓ* and $\ell^{\prime }$, respectively. Then, $\left\{e_{1},\ldots ,e_{n};Je_{1},\ldots ,Je_{n}\right\}$ and $\{e_{1}^{\prime },\ldots ,e_{n}^{\prime };$ $Je_{1}^{\prime },\ldots ,Je_{n}^{\prime }\}$ are both symplectic and orthogonal bases of $\left(R^{2n},\sigma \right)$. The automorphism *U* of $R^{2n}$ taking the first basis to the second is thus in Sp(n)∩O(2n,R) and we have $\ell^{\prime }=U\ell$.

**Lemma** **A1.**
*Let ℓ∈Lag(n). There exist real n×n matrices A,B with rank(A,B)=n and $A^{T}B=B^{T}A$ and $AB^{T}=BA^{T}$ such that*

$$\ell =\{\left(x,p\right)\in R^{2n}:Ax+Bp=0\}.$$

**Proof.** It is a straightforward consequence of the transitivity of the action (A7) of U(n) on Lag(n). □

Note that in particular, the equation p=Mx defines a Lagrangian plane *ℓ* such that $\ell \cap (0\times R^{n})=0$ if and only if *M* is symmetric and detM≠0.
